# Development and validation of a machine learning-augmented algorithm for diabetes screening in community and primary care settings: A population-based study

**DOI:** 10.3389/fendo.2022.1043919

**Published:** 2022-11-28

**Authors:** XiaoHuan Liu, Weiyue Zhang, Qiao Zhang, Long Chen, TianShu Zeng, JiaoYue Zhang, Jie Min, ShengHua Tian, Hao Zhang, Hantao Huang, Ping Wang, Xiang Hu, LuLu Chen

**Affiliations:** ^1^ Department of Endocrinology, Union Hospital, Tongji Medical College, Huazhong University of Science and Technology, Wuhan, China; ^2^ Hubei provincial Clinical Research Center for Diabetes and Metabolic Disorders, Wuhan, China; ^3^ Department of Cardiovascular Surgery, Union Hospital, Tongji Medical College, Huazhong University of Science and Technology, Wuhan, China; ^4^ Department of Computer Science and Technology, Tsinghua University, Beijing, China; ^5^ Yiling Hospital, Yichang, China; ^6^ Precision Health Program, Department of Radiology, College of Human Medicine, Michigan State University, East Lansing, MI, United States

**Keywords:** diabetes, screening, ML-augmented algorithm, community and primary care, health economic evaluation

## Abstract

**Background:**

Opportunely screening for diabetes is crucial to reduce its related morbidity, mortality, and socioeconomic burden. Machine learning (ML) has excellent capability to maximize predictive accuracy. We aim to develop ML-augmented models for diabetes screening in community and primary care settings.

**Methods:**

8425 participants were involved from a population-based study in Hubei, China since 2011. The dataset was split into a development set and a testing set. Seven different ML algorithms were compared to generate predictive models. Non-laboratory features were employed in the ML model for community settings, and laboratory test features were further introduced in the ML+lab models for primary care. The area under the receiver operating characteristic curve (AUC), area under the precision-recall curve (auPR), and the average detection costs per participant of these models were compared with their counterparts based on the New China Diabetes Risk Score (NCDRS) currently recommended for diabetes screening.

**Results:**

The AUC and auPR of the ML model were 0·697and 0·303 in the testing set, seemingly outperforming those of NCDRS by 10·99% and 64·67%, respectively. The average detection cost of the ML model was 12·81% lower than that of NCDRS with the same sensitivity (0·72). Moreover, the average detection cost of the ML+FPG model is the lowest among the ML+lab models and less than that of the ML model and NCDRS+FPG model.

**Conclusion:**

The ML model and the ML+FPG model achieved higher predictive accuracy and lower detection costs than their counterpart based on NCDRS. Thus, the ML-augmented algorithm is potential to be employed for diabetes screening in community and primary care settings.

## Introduction

Diabetes is highly prevalent among adults worldwide and the prevalence has been expanding in developing societies including China and India, consequently increasing the incidence of multiple diabetes complications including cardiovascular disease, retinopathy, kidney disease, neuropathy, blindness, and lower-extremity amputation (1). These complications result in increased morbidity and mortality and impose a heavy economic burden on patients and their health care systems globally (2).

Fortunately, more evidence indicates that early diagnosis and opportune management of diabetes can prevent or delay diabetic complications and dramatically alleviate its related morbidity, mortality, and economic burden (3–5). Diabetes screening is crucial in the early detection and diagnosis of diabetes. Notably, it is estimated that 50·1% of adults (231·9 million) living with diabetes were undiagnosed around the world in 2019, since diabetes usually has a long asymptomatic phase and blood glucose testing is not always available or accessible in communities (1, 6). Additionally, fasting plasma glucose (FPG) is routinely used for diabetes screening but there are a large number of patients with isolated post-load hyperglycemia, which makes a large proportion of patients with missed diagnosis of diabetes (around 38% in our previous study) (7). Moreover, further confirmatory tests such as OGTT (oral glucose tolerance test) or HbA1c cost more and are time-consuming (8). Therefore, it would be extremely useful for timely diagnosis and treatment of diabetes to develop an easy-to-use, very convenient and accessible, and economical screening system with superior sensitivity and specificity to screen out the residents with a high risk of diabetes in the community for further confirmatory test of OGTT, and sift out the individuals likely to have isolated post-load diabetes in primary care for the confirmatory test to detect diabetes.

Risk-based screening for diabetes is currently recommended by ADA (America Diabetes Association) and the Chinese Diabetes Society (CDS) (9, 10). The ADA recommends the use of the scoring table of ADA risk test (ADART), and CDS recommends the use of the New Chinese Diabetes Risk Score (NCDRS) to screen out high-risk individuals for the further confirmative test. These two scoring tables developed by Logistic regression (LR) based on easy-to-accessible non-laboratory characteristics are of great help in clinical practice for diabetes screening (8, 11). Notably, there is increasing evidence indicating that machine learning (ML) methods are able to predict relationships between input and output by extracting information from a larger number of complex variables and have shown accurate predictive ability (12, 13). It has been increasingly introduced in health service research and has shown a better level of prediction than traditional statistical approaches in several domains (14–16). Recently, it is reported by Vangeepuram, N., et al. that Some ML-based classifiers derived from the National Health and Nutritional Examination Survey (NHANES) dataset in the United States performed comparably to or better than the screening guideline in identifying preDM/DM youth (17). Herein, we hypothesized that the ML-based algorithm has the potential to develop predictive models for diabetes screening and be helpful to screen out people with a high risk of diabetes in the community and individuals likely to have isolated post-load diabetes in primary care more accurately and conveniently for further confirmation and diagnosis opportunely, which would outperform standard risk-scoring algorithms for screening diabetes and increase the cost-effectiveness of detection by reducing the false-positive screening rate.

In this study, we aimed to develop and validate a ML-augmented algorithm for diabetes screening in community and primary care settings with data from a population-based study in China.

## Methods

### Study design, participants, and data collection

**Figure 1:**
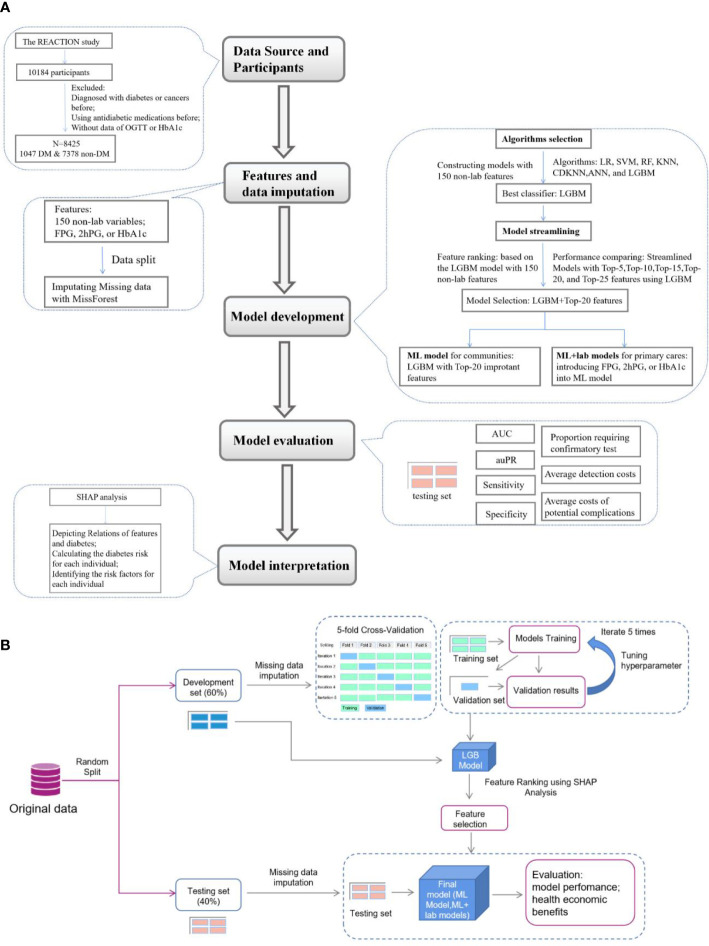
Flow diagram of the research. **(A)**, Overview of the study. **(B)**, Details of feature selection and model evaluation REACTION, Risk Evaluation of Cancers in Chinese diabetic Individuals; N, the numbers of participants involved in this study; DM, person with diabetes mellitus; SHAP analysis, Shapley Addictive exPlanations analysis; LR, Logistic Regression; SVM, Support Vector Machine; RF, Random Forest; KNN, K-nearest neighbors algorithm; CDKNN, Centroid-Displacement-based-k-NN; ANN, Artificial Neuron Network; LGBM, light gradient boosting machine; AUC, area under the receiver operating characteristic curve (ROC); auPR, area under the precision versus recall curve.

The data analyzed in the present investigation were obtained from the Hubei Yiling center of the Risk Evaluation of cAncers in Chinese diabeTic Individuals: a lONgitudinal (REACTION) study performed from October 2011. Detailed information and the study design of the REACTION study were described previously (18). A total of 10184 eligible subjects were enrolled in this study. Data collection was performed by the trained staff and a questionnaire was completed as described previously for gathering information on demographic characteristics, data on lifestyle, and medical history (7). Weight, height, waist circumference (WC), hip circumference (HC), blood pressure, and resting pulse rate (RPR) were measured according to standard protocols. Body mass index (BMI), waist-to-hip ratio (WHR), and waist-to-height ratio (WHtR) were calculated as described previously (19). Participants without a self-reported history of diabetes were provided with a standard 75 g glucose solution, and blood sampling was conducted at 0 and 2 h after administration. Plasma glucose was measured using the glucose oxidase method. HbA1c was tested using finger capillary whole blood by high-performance liquid chromatography. Participants who had been diagnosed with cancers or diabetes before or using antidiabetic agents, or whose data on FPG, 2hPG or HbA1c were missing were excluded and 8425 participants were included in this analysis. The data collected were analyzed and the flow chart of this study was shown in **Figure 1**.

The present study complies with the Declaration of Helsinki, and all procedures were approved by the Ethics Committee of Tongji Medical College, Huazhong University of Science and Technology. Written, informed consent was obtained from all the participants.

### Outcome

Diabetes was diagnosed based on measurement of fasting blood glucose level, or oral glucose tolerance testing as recommended by CDS when the study was performed, which included the following: a FPG level of ≥7·0mmol/l, or OGTT-2h post-load plasma glucose (2hPG) level of ≥11·1 mmol/l.

### Predictors and data processing

150 non-laboratory (demographics and anthropometric) and three laboratory features (FPG, 2hPG, HbA1c), were included in the analysis after excluding features with more than 20% missing data as described previously (20, 21). MissForest was used to impute missing values for the features with less than 20% missing data as described previously (22). Data were divided into a development set (60%) and a testing set (40%) and the imputation was trained in the development and applied to the testing set to avoid data leakage. A complete case sensitivity analysis was performed to compare the difference before and after imputation of missing data (**Supplementary Tables 1, 2**). The development set was used for training and validating models. The testing set was blind to the training, hyperparameter tuning, and feature selection, and were only used to evaluate the performance and health economic benefits of models.

### Models development by machine learning for the community and primary care

Logistic Regression (LR), Support Vector Machine (SVM), Random Forest (RF), K-nearest neighbors algorithm (KNN), Centroid-Displacement-based-k-NN (CDKNN) (23), Artificial Neuron Network (ANN), and Light Gradient Boost Machine (LGBM) were preliminarily tested as the classification algorithms to develop the predictive models for diabetes screening with 150 non-laboratory features. TPE (Tree of Parzen Estimators) was used to tune hyperparameters and improve model prediction capability (24). The hyperparameters were reported in **Supplementary Table 3**. For internal validation, 5-fold cross-validation was used as reported previously (25–27). In detail, we randomly and equally split the development set into 5 folds using the random.shuffle() function of the Numpy library in Python, and for each iteration, we employed four folds to train the models, and the remaining fold to validate the models independently. The algorithm that had the best predictive ability was selected to develop the predictive models in this study.

Shapley Additive Explanations (SHAP) analysis (28) was employed to interpret the results and analyze the importance of (the contribution of a feature value to the difference between the actual prediction and the mean prediction is the estimated Shapley value) of 150 non-laboratory features of the model with the best performing algorithm (LGBM). Five streamlined models were developed using 5, 10, 15, 20, 25 features of top-ranked importance among all the non-laboratory features with LGBM to simplify the predictive model for practice. The streamlined model with the best predictive power and the least number of features was selected and noted as the ML model for diabetes screening in community care.

**Figure 2:**
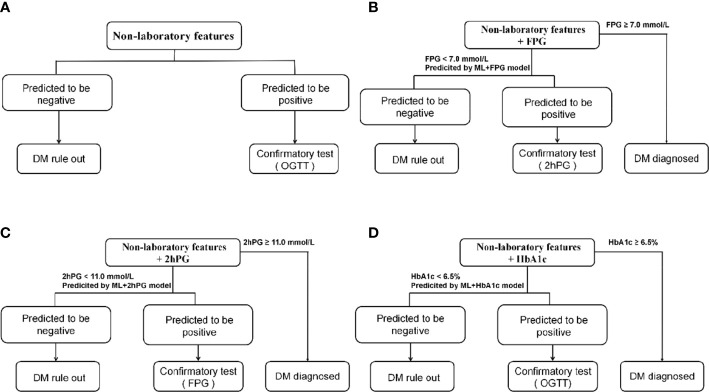
Diabetes detection procedures of models. **(A)**, Detection procedures of ML model and NCDRS. **(B)**, Detection procedures of ML+FPG model and NCDRS+FPG model. **(C)**, Detection procedures of ML+2hPG model and NCDRS+2hPG model. **(D)**, Detection procedures of ML+HbA1c model and NCDRS+HbA1c model. OGTT in a and d included fasting plasma glucose and 2h post-load plasma glucose. NCDRS, New Chinese Diabetes Risk Score; DM, diabetes mellitus; OGTT, oral glucose tolerance test; FPG, fasting plasma glucose; 2hPG, OGTT-2h post-load plasma glucose, HbA1c, glycated hemoglobin A1c.

A large number of individuals with seemingly normal levels of FPG (FPG<7.0mmol/L) or 2hPG (2hPG<11.0mmol/l) are potentially diabetes patients and too many patients would not be diagnosed opportunely using only one testing mentioned above. However, these seemingly normal levels of FPG, 2hPG, or HbA1c are likely to be useful in the prediction of diabetes. Thus, we tried to develop the ML+lab models by introducing one test (seemingly normal FPG, 2hPG, or HbA1c) in the hope of getting an efficient, cost-effective, and convenient screening model in primary care to decrease the missed diagnoses of diabetes and related costs (**Figure 2**). That is, the ML+FPG, ML+2hPG or ML+HbA1c models predicted isolated post-load diabetes (identified using 2hPG) by introducing seemingly normal FPG levels, and isolated fasting diabetes (identified using FPG) by seemingly normal 2hPG levels and diabetes (identified using FPG and 2hPG) by normal HbA1c, respectively.

The ML model was compared with the ADART and NCDRS. The ML+lab models were compared with the ML model and with the corresponding NCDRS+lab models developed by combining FPG, 2hPG, or HbA1c and the NCDRS.

### Model performance evaluation

We used the area under the receiver operating characteristic curve (AUC), the area under the precision-recall curve (auPR), sensitivity, specificity, and precision to evaluate model predictive ability. True positive (TP), true negative (TN), false positive (FP), false negative (FN) cases, and negative cases were calculated for further analysis. Sensitivity (recall), a measure of the ability of the model to identify diabetes, was defined as TP/(TP+FN). Specificity, the proportion predicted to be negative among the non-diabetes population, was calculated by TN/(FP+TN). Precision was defined as TP/(TP+FP). AUC was calculated from the curve of sensitivity against 1-specificity and auPR was calculated from the precision-recall curve.

### Health economic evaluation

Individuals at high risk of diabetes are recommended to perform the confirmatory test in clinical practice according to guidelines (9, 10). Therefore, we attempted to identify the risk of individuals with the ML model, ML+lab models, NCDRS, or NCDRS+lab models in the present study, to screen out those with high risk for the further confirmatory test. The individuals requiring confirmatory test in this study were those who were predicted to be positive and screening tests of FPG < 7·0 mmol/l or 2hPG < 11·0 mmol/l (if available) in the testing set. The proportion requiring confirmatory test was calculated by dividing the numbers of individuals requiring confirmatory test by the number of participants in the testing set (**Figure 2**; **Supplementary Table 4**).

The costs associated with screening tests (FPG, 2hPG, or HbA1c in the ML/NCDRS+lab models) and confirmatory tests were made up of medical costs and non-medical costs (e.g transportation costs). Medical costs (CNY) for FPG, 2hPG, OGTT, and HbA1c were 9·89, 23·56, 33·45, and 84·16, respectively as described previously (7, 29), and non-medical costs (CNY) calculated based on the report described previously were 8·3, 27·5, 27·5, and 8·3 for FPG, 2hPG, OGTT, and HbA1c, respectively (30). A further confirmatory test is required after the diabetes screening, a process known as diabetes detection. The average detection costs per participant of using predictive models as a screening strategy were calculated by dividing the sum of the costs associated with screening and confirmatory test by the number of participants in the testing set (**Supplementary Table 4**).

In view that early diagnosis and opportune management of diabetes can prevent or delay diabetic complications, in the present study we tried to assess the potential complication costs, i.e. costs of false negative were assessed by estimating the increased costs from complications not prevented by the lack of timely and appropriate intervention for diabetes (31). The potential complication costs per individual with diabetes per year in China were estimated after adjusting for medical cost differences based on a previous study (31), ranging from (CNY) 341 to 567, 1302 to 2555, 2802 to 5611, 4428 to 8212, and 5258 to 9132 over 5, 10, 15, 20, and 25 years, respectively. The average costs of potential complication per participant of using predictive models as a screening strategy were calculated by dividing the sum of potential complication costs of all cases of FN by the number of participants in the testing set (**Supplementary Table 5**) (7).

### Model interpretation

SHAP analysis was employed for the interpretation of the ML model. In detail, SHAP values were calculated for the top-10 features and converted to relative risk (RR) of diabetes to explore the relationships between features and diabetes risk as reported in previous studies (32). In addition, two cases of TP were selected randomly as examples to demonstrate practically how the ML model works. In the demonstration, their respective key predictors were classified respectively, and the importance of their respective predictors was assessed by calculating SHAP values, and predictive risk of diabetes was evaluated individually by summing their SHAP values.

### Statistical analysis

Data of participants were presented as medians (IQRs) for continuous variables and numbers (proportions) for categorical variables. These data were tested for normality using the Kolmogorov-Smirnov test. The Kruskal-Wallis test was used to compare continuous variables (skewed variables) and the chi-square test was used to compare categorical variables and the Delong test was used to compare AUC. A 2-sided P value <0·05 was considered statistically significant. Data were analyzed using Python 3·7 and SPSS 20·0. MissForest, LR, SVM, and RF were implemented with the ML library “sklearn” (33), and the ANN was implemented with PyTorch. LGBM referred to https://lightgbm.readthedocs.io/. SHAP analysis referred to http://github.com/slundberg/shap (28).

## Results

### Characteristics of participants

In the present study, 1047 (12·4%) were diagnosed with diabetes. Principally, the age, resting pulse rate, blood pressures, weight, BMI, WC, WHR, WHtR, FPG, 2hPG, and HbA1c were higher, and the amount of physical activity and alcohol consumption was lower (P<0·05) in participants with diabetes compared to individuals without diabetes, while no significant differences were observed in sex and numbers of smoker (P>0·05) (**Table 1**).

**Table 1:** Characteristics of participants.

	Total (n=8425)	Non-DM (n=7378)	DM (n=1047)	P
Age, years^a^	54·0 (47·0-60·0)	54·0 (47·0-60·0)	57·0 (50·0-63·0)	0·000
Sex, n (%)^b^				0·090
Male	2989 (35·5)	2593 (35·1)	396 (37·8)	
Female	5436 (64·5)	4785 (64·9)	651 (62·2)	
Smoke, n (%)^b^				
Current smoker	1621 (19·2)	1427 (19·3)	194 (18·5)	0·789
Past smoker	353 (4·2)	307 (4·2)	46 (4·4)	
Never smoker	6451 (76·6)	5644 (76·5)	807 (77·1)	
Drink, n (%)^b^				0·006
Current drinker	1088 (12·9)	921 (12·5)	167 (16·0)	
Past drinker	203 (2·4)	176 (2·4)	27 (2·6)	
Never drinker	7134 (84·7)	6281 (85·1)	853 (81·5)	
Physical activity, n (%)^b^				0·044
inactive	7595 (90·1)	6633 (89·9)	962 (91·9)	
active	830 (9·9)	745 (10·1)	85 (8·1)	
SBP, mmHg^a^	135·0 (122·5-150·7)	134·0 (122·0-149·3)	141·0 (128·0-157·5)	0·000
DBP, mmHg^a^	79·0 (71·0-87·0)	78·5 (71·0-86·7)	81·7 (74·0-90·3)	0·000
RPR, bpm^a^	80·0 (72·3-88·0)	79·0 (72·0-87·0)	85·0 (77·0-95·3)	0·000
Weight, kg^a^	57·0 (50·7-63·0)	56·5 (50·5-63·0)	58·5 (52·0-65·0)	0·000
Height, m^a^	1·56 (1·52-1·62)	1·56 (1·52-1·62)	1·56 (1·51-1·62)	0·306
WC, cm^a^	78·0 (72·0-84·0)	77·5 (71·0-84·0)	80·3 (74·0-87·0)	0·000
HC, cm^a^	90·0 (86·0-95·0)	90·0 (86·0-95·0)	91·0 (87·0-96·0)	0·005
BMI, kg/m2^a^	23·1 (21·0-25·4)	23·0 (20·9-25·2)	23·9 (21·6-26·3)	0·000
WHR, cm/cm^a^	0·86 (0·81-0·90)	0·86 (0·81-0·90)	0·89 (0·83-0·93)	0·000
WHtR, cm/m^a^	0·50 (0·46-0·54)	0·49 (0·46-0·54)	0·52 (0·47-0·55)	0·000
FPG, mmol/l^a^	5·7 (5·3-6·1)	5·6 (5·3-6·0)	7·1 (6·2-8·0)	0·000
2hPG, mmol/l^a^	6·9 (5·8-8·3)	6·6 (5·7-7·8)	12·2 (10·3-14·9)	0·000
HbA1c, %^a^	5·5 (5·2-5·8)	5·5 (5·2-5·8)	6·0 (5·6-6·6)	0·000

a Data (continuous variables non-normally distributed) are expressed as medians (interquartile range).

b Data (categorical variables) are expressed as N (%).

DM, diabetes mellitus; SD, standard deviation; n (%), number of participants and percentage over the total number of participants; SBP, systolic blood pressure; DBP, diastolic blood pressure; RPR, resting pulse rate; WC, waist circumference; HC, hip circumference; BMI, body mass index; WHR, waist-to-hip ratio; WHtR, waist-to-height ratio; FPG, fasting plasma glucose; 2hPG, OGTT-2h post-load plasma glucose; HbA1c, glycated hemoglobin A1c.

### Development and interpretation of the ML model for community care

**Figure 3:**
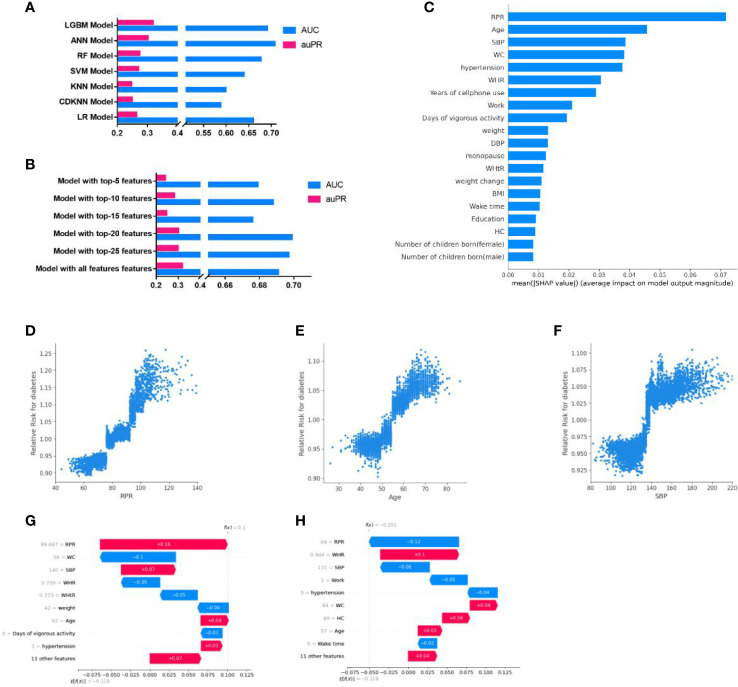
Development and interpretation of the ML model. **(A)**, Compared the AUC and auPR among the models with seven algorithms. **(B)**, Compared the AUC and auPR among streamlined models and the model with all features. **(C)**, Importance of the features in the ML model. The input features on the y-axis are ordered by descending importance and the values on the x-axis indicate the mean impact of each feature on model output magnitude based on SHAP analysis. **(D–F)**, The relative risk of diabetes of RPR **(D)**, Age **(E)**, and SBP **(F)**. Each point represented the predicted relative diabetes risk of each individual. The relative risk value above 1·0 for specific features represent an increased risk of diabetes. **(G-H)**, Personalized risk prediction for two cases from the validation set of the ML model. The y-axes represent the input features ordered by descending importance. f(x) is the personalized model output for a participant. If f(x) is larger than e[f(x)], the patient has a higher risk of diabetes relative to the background population. Each arrow represents how a specific feature increases (red) or decreases (blue) the participant’s risk for diabetes. LGBM, light gradient boosting machine; ANN, Artificial Neuron Network; RF, Random Forest; SVM, Support Vector Machine; LR, Logistic Regression; KNN, K-nearest neighbors algorithm; CDKNN, Centroid-Displacement-based-k-NN; RPR, resting pulse rate; WHR, waist-to-hip ratio; SBP, systolic blood pressure; BMI, body mass index; WC, waist circumference; WHtR, waist-to-height ratio; DBP, diastolic blood pressure; HC, hip circumference.

Our results indicated that the predictive model developed by LGBM for diabetes screening seemingly had the best performance, with the highest auPR of 0·319 [95%CI, 0·267-0·386] and AUC of 0·691[95%CI, 0·641-0·733] (**Figure 3A**; **Supplementary Table 6**), among the models created by the seven ML algorithms (LR, KNN, CDKNN, SVM, LR, ANN, LGBM). To simplify the used features in the model, five streamlined models were developed with LGBM using the top-5, top-10, top-15, top-20, and top-25 features, respectively, according to the feature importance. Among these models, the AUC (0·699 [95%CI, 0·663-0·736]) of the streamlined model with top-20 features was highest (**Figure 3B**). While the auPR of the streamlined model with top-20 features (0·301[95%CI, 0·220, 0·390]) was closest to that of the model with all features. The model with top-20 features seemed a fairly convenient and accurate model and was adopted for the subsequent analysis, noted as the ML model in the present study.

The top-20 important features of the ML model included resting pulse rate (RPR), Age, systolic pressure (SBP), waist circumference (WC), work status, WHR, etc (**Figure 3C**). The most important predictors associated with the predictive power of the model were RPR, age, and SBP, which were positively associated with RR of diabetes (when RPR was higher than 78, age was higher than 52 years, or SBP exceeded 135, the RR of diabetes >1) (**Figures 3D–F**). The RRs of diabetes for other top-10 important features were shown in **Supplementary Figure 1**. In addition, SHAP analysis performed by taking two cases, for example, indicated that individualized important predictors were identified for case1(including RPR, SBP, age, etc) and case2 (including WHR, WC, and age, etc). The magnitudes of these predictors were also assessed (**Figures 3G, H**).

### Comparison of the predictive ability between ML and ML+lab models with their corresponding counterparts

**Figure 4:**
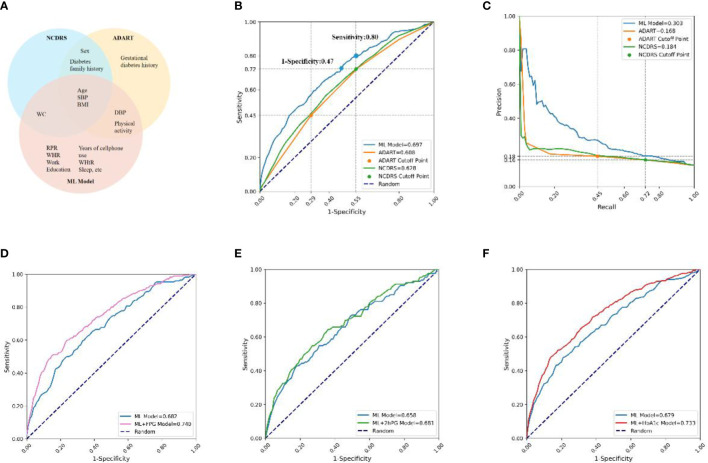
Comparisons of features and prediction performance between ML and ML+lab models with their corresponding counterparts. **(A)**, Compared features between the ML model and ADART and NCDRS. **(B, C)**, Compared the ROC **(B)** and PR **(C)** curves between the ADART, NCDRS, and the ML model. vs AUC (ML model), P< 0·05; the cutoff points of ADART and NCDRS recommended by ADA and CDS (Risk score ≥ 5 and ≥ 25, respectively) were plotted as two points in ROC and PR curve; the corresponding horizontal and vertical coordinate values of these two points are marked on the coordinate axis. **(D–F)**, Compared the ROC curves between the ML model with ML+FPG model **(D)** in individuals with seemingly normal FPG levels (P< 0·05), ML+2hPG **(E)** model in individuals with seemingly normal 2hPG levels (P> 0·05), and ML+HbA1c model **(F)** in individuals with seemingly normal HbA1c levels (P< 0·05). ADA, America Diabetes Association; CDS, Chinese Diabetes Society; ADART, ADA risk test; NCDRS, New Chinese Diabetes Risk Score.

The 150 features of our predictive model included all the features used in ADART and NCDRS, and the top-20 features of the ML model comprised most of the features used in ADART (4 of 7) and NCDRS (4 of 6). In addition, our results indicated that 15 features, which were not included in ADART and NCDRS, were also very important in the ML model (**Figure 4A**).

The AUC was 0·697 in the ML model in the testing set, which was higher than that in the ADART (0·608) and NCDRS (0·628). The auPR of the ML model (0·303) was also higher than that of the ADART (0·168) and NCDRS (0·184) (**Figures 4B, C**, **Supplementary Table 7**). Remarkably, the analysis in our study indicated that the NCDRS had higher AUC and auPR compared with the ADART. Therefore, we chose to perform further comparisons between our models and NCDRS on sensitivity, specificity and health economic evaluation. Our analysis indicated that the sensitivity and specificity of the NCDRS were 0·72 and 0·45 respectively using the cutoff recommended by CDS, which is considered to be optimal in the Chinese population (10). The ML model had higher sensitivity (0·80 vs 0·72) when it had the same specificity as NCDRS (0·45). Likewise, The ML model had higher specificity (0·53 vs 0·45) when it had the same sensitivity as NCDRS (0·72).

Our results indicated that 41% of diabetes patients have seemingly normal FPG (FPG<7·0mmol/l), 28% of them have seemingly normal 2hPG (2hPG<11·0mmol/l), and only 31% of them have a combination of increased FPG (FPG ≥7·0mmol/l) and 2hPG (2hPG ≥11·0mmol/l) levels, which are both higher than the diagnostic criteria (**Supplementary Figure 2**), implying that using one testing of FPG or 2hPG only would lead to numerous missed diagnosis of diabetes.

The results showed that the AUC of the ML+FPG model was 0·740, which was higher by 8.5% compared with the ML model (0·682) in individuals with seemingly normal FPG levels (P<0·05). Likewise, the ML+HbA1c model had a 8.0% increase compared to the ML model in individuals with seemingly normal HbA1c levels (P<0·05). While the AUC of ML+2hPG model had no statistical difference with that of the ML model in individuals with seemingly normal 2hPG levels (P>0·05) (**Figures 4D–F**).

Moreover, the AUC of the ML+FPG model was higher than that of the NCDRS+FPG model in seemingly normal FPG individuals (P<0·05). Likewise, the AUC were higher in the ML+2hPG and ML+HbA1C models compared with their corresponding counterparts based on NCDRS (**Supplementary Figure 3**).

### Comparison of the health economic costs between ML and ML+lab models with their corresponding counterparts

**Figure 5:**
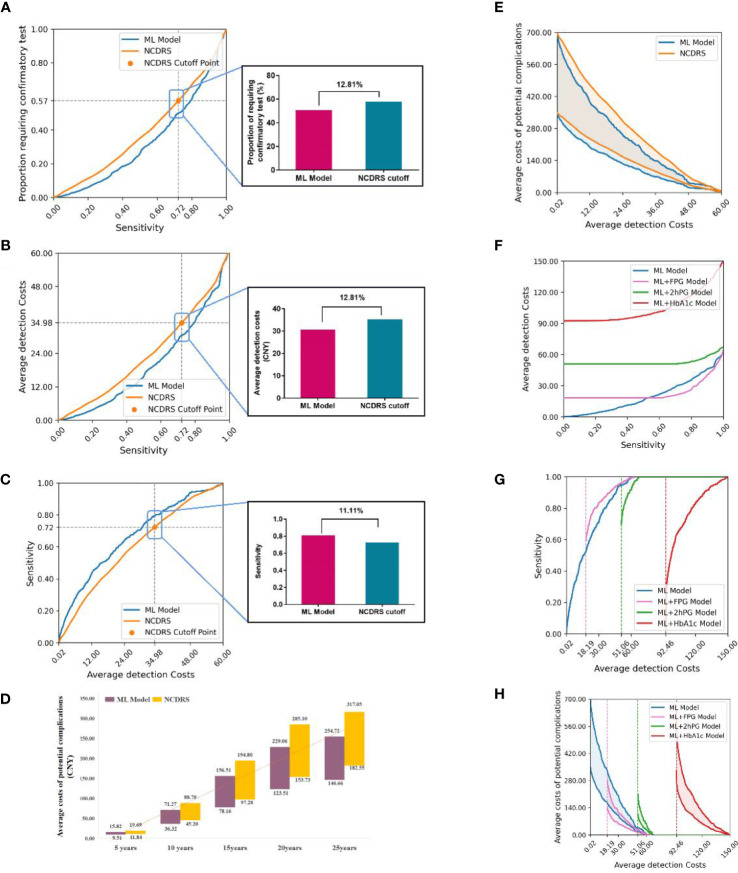
Comparison the health economic costs between ML and ML+lab models with their corresponding counterparts. **(A-B)**, Compared the proportion requiring confirmatory test **(A)** and average detection costs **(B)** between the ML model and NCDRS at a different level of sensitivity. **(C)**, Compared the sensitivity between the ML model and NCDRS at a different level of average detection costs. **(D)**, Compared the average costs of potential complications in the next 5-25 years between the ML model and NCDRS when the average detection costs was ¥34·98 (When using the cutoff point of the CDS guideline, the average detection costs of NCDRS were ¥34·98). **(E)**, Compared the average costs of potential complications between the ML model and NCDRS at a different level of average detection costs. **(F)**, Compared the average detection costs at a different level of sensitivity between the ML model with ML+lab models. g-h, Compared the sensitivity **(G)** and the average costs of potential complications for 15 years **(H)** at a different level of average detection costs between the ML model with ML+lab models. The number on the horizontal line **(A-C)** represents the relative reduction of the proportion requiring confirmatory test, average detection costs, and increase of sensitivity of the ML model compared to the NCDRS. The numbers in the bar chart **(D)** represent the range of average costs of potential complications. The dotted lines in **(G, H)** represent the basic average detection costs of the ML+lab models which are the costs of screening test of these models. Shaded areas represent the range of average costs of potential complications for 15 years.

Our results indicated that the proportion requiring confirmatory test and the average detection costs per participant using the ML model were lower than those using NCDRS at any sensitivity. The proportion requiring confirmatory test was 57·96% and 50·07% in NCDRS and our ML model when they had the same sensitivity of 0·72, and consequently, their average detection costs per participant were ¥34.98 and ¥30·41 respectively in the analysis of the present study. These results suggested that the proportion requiring confirmatory test and the average detection costs per participant were both lower by 12.81% in our ML model than those in the NCDRS (**Figures 5A, F, B**). In addition, the ML model had higher sensitivity (0·80 vs 0·72) when it had the same average detection costs as NCDRS (¥34.98) and at any average detection costs (**Figure 5C**). Furthermore, the average costs of potential complications per person per year of the ML model (ranged from ¥78.16 to ¥156.51 for 15 years) decreased by 19.7% compared with the NCDRS (ranged from ¥97.28 to ¥194.8 for 15 years) when it had the same average detection costs as NCDRS (¥34.98) (**Figure 5D**). Likewise, the ML model had a lower average costs of potential complications for 15 years per participant than the NCDRS at any average detection costs (**Figure 5E**).

The average detection costs per participant were lower in the ML+FPG model and were higher in the ML+2hPG and ML+HbA1c models compared with the ML model (**Figure 5F**). The sensitivity was higher and the average costs of potential complications per participant were lower in the ML+FPG compared with the ML model. However, the sensitivity was lower and average costs of potential complications for 15 years per participant were higher in ML+2hPG and ML+HbA1c models compared with the ML model (**Figures 5G, H**).

Moreover, the average detection costs per participant decreased in the ML+FPG model compared with the NCDRS+FPG model. The sensitivity was higher and the average costs of potential complications per participant were lower in the ML+FPG model than in the NCDRS+FPG model. Likewise, the sensitivity was higher, and the average detection costs per participant as well as average complication costs per participant were lower in the ML+2hPG and ML+HbA1C models compared with their corresponding counterparts based on NCDRS (**Supplementary Figure 4**).

## Discussion

In the present study, we attempted to develop an easy-to-use ML-augmented prediction model for diabetes screening using population-based data from China. Our analysis indicated that the ML model developed with non-laboratory features for community care had superior predictive accuracy, and could lower average detection costs per participant by 12.81% compared with the NCDRS even if it had the same sensitivity as NCDRS. Additionally, the ML+lab models which were developed for primary care by adding laboratory tests to the ML model, had even better predictive accuracy. Remarkably, the ML+FPG model had considerable accuracy and lower average detection costs compared with the ML model.

In recent years, there is a growing body of evidence indicating that ML seems very promising in risk predictions for disease (32, 34, 35). In this study, our ML model had higher sensitivity, specificity, AUC, and auPR compared to the NCDRS, implicating that our ML model seemed to have superior predictive accuracy. As a data-driven method, ML is widely considered to be able to detect complex nonlinear relationships and probable interactions between variables and outcomes (36). Additionally, ML is capable of mining more information from big data compared to Logistic Regression and consequently, more predictors could be handled in predictive models developed by ML (37). Remarkably, there is a rich library of available machine learning methods used for developing models to deal with a specific problem, and it is crucial to select an appropriate ML method to improve the performance of the models (4). Recent studies indicate that diabetes prediction models developed based on images, electronic health records, or structured data obtained from their societies, using machine learning algorithms such as Decision Tree, Naive Bayes, SVM, ANN, etc., achieve superior performance and demonstrate their potential to be helpful for diabetes screening (38, 39). We employed seven ML algorithms for diabetes screening using data from our population-based study in China, including LGBM, ANN, SVM, RF, KNN, CDKNN and LR, which are reported to have good performances in developing predictive models with high accuracy in recent studies (40–44). Our results indicated that the LGBM model had the best predictive accuracy among the models developed with the five algorithms in our societies. It is considered that LGBM acts as the state of the art in developing predictive models for tabular data (32), and a very recent study indicates that Gradient Boost Machine (GBM) performed better than logistic regression (LR), classification, and regression tree (CART), artificial neural networks (ANN), support vector machine (SVM) and random forest (RF) (45), which might be important explanations of our results that the ML model seemed to have higher predictive accuracy compared with NCDRS. Noteworthy, we tested the ensemble algorithm that combined the classifiers of LR, RF, ANN, and LGBM with the same features as the ML model for predicting diabetes. The results indicated that the ensemble algorithm had a slightly higher AUC than LGBM, while there was no statistically significant difference (**Supplementary Figure 5**, P>0·05). New algorithms for designing accurate and effective models for diabetes screening still need further investigation in the future.

Notably, we analyzed the performance of our models with methods recommended in the current guideline for screening diabetes in adults and the results indicated that our models seemed to perform better. Similarly, a very recent study by Vangeepuram, N., et al. indicated that some ML-based classifiers derived from the NHANES dataset in the United States performed comparably to or better than the screening guideline in identifying preDM/DM youth, which is another important evidence that ML model seemed to have better performance compared with recommendations of the current guidelines (17). In addition, the diabetes prediction models constructed by Binh P.Nguyen et,al and Wei et,al achieved good prediction performance using 1321 features (including various laboratory tests) and environmental chemical exposures that are not routinely tested in daily life, respectively (46, 47). Our ML model used 20 features that are easily available in daily life and clinical practice in order to make the prediction model more convenient for use in the community and primary care settings. Additionally, we evaluated the proportion requiring confirmatory test and the average detection costs to assess the health economic benefits of the ML models in practice, which is a new attempt compared to previous ML diabetes prediction studies (4, 17, 45, 47–53).

Our analysis demonstrated that the ML model could save the average detection costs per participant by 12.81% compared with NCDRS, without sacrificing sensitivity. The cost saving was mainly attributed to the higher predictive accuracy, which consequently enhanced specificity and decreased the false-positive rate in the ML model, resulting in a considerable decline in the number of confirmatory test and related costs (54). In addition, the ML model could lower the average costs of potential complications per participant by 19.7% compared with NCDRS in this study, which might be ascribed to the increase in predictive accuracy and sensitivity, as well as the decreases in misdiagnosis in the ML model compared with NCDRS, lessening the incidence of complications and corresponding costs for the lack of timely detection and intervention of diabetes.

Remarkably, the features used in our ML model are readily obtainable information, enabling residents to estimate their risk of diabetes in communities without seeing their doctors for any medical examinations or laboratory tests. Most of the variables (RPR, WC, sleep-associated issues, etc) in the ML model were routinely collected in clinical practice in China, and those variables involved in the ML models which are not routinely collected by medical practitioners (education, work status, etc) can be quickly obtained by easy-to-use questionnaires, which are different from those variables in diabetes predictive models including tongue features and environmental chemical exposure developed in very recent studies (47, 51, 52), and might be a little bit more convenient in practice. Thus, the ML model is potentially beneficial for diabetes screening in communities and primary care settings with greater convenience and accessibility, as well as higher predictive accuracy and less cost as mentioned above. Moreover, the ML model could be hopefully further developed and presented in open and accessible web pages to make it easier and more available for residents to evaluate the risk of diabetes in communities, and the information increasingly inputted in the ML model for risk prediction is very helpful to improve the performance of the model in turn, which is an advantage of ML (28).

FPG, 2hPG, or/and HbA1c are important screening and diagnostic tests of diabetes (9). It should be noted that all these tests are not usually employed for detecting diabetes in the same individuals at the same visit in clinical practice and one testing of them only may lead to underdiagnosis of diabetes (9). Actually, there are a large number of diabetes with seemingly normal FPG or 2hPG levels since the concordance between the FPG and 2hPG tests is imperfect (55), which is also observed in our present investigation. It is reported that seemingly normal FPG, 2hPG, and HbA1c were important predictors of future diabetes (56–58). Thus, we tried to develop the ML+lab models by introducing FPG, 2hPG, or HbA1c with seemingly normal levels for diabetes screening in primary care settings. Our data indicated that introducing FPG, 2hPG, or HbA1c in individuals with normal levels of these testing increased the efficiency and accuracy of the predictive models. Moreover, the predictive accuracy of ML+FPG and ML+HbA1c models in individuals with normal levels of these testing seemed higher than that of ML model in all participants. These results suggested that it seemed practical and beneficial to adapt ML+lab models to screen out diabetes patients with seemingly normal FPG, 2hPG or HbA1c. That ML could mine maximal information from the simple features might be an important explanation for the effectiveness of our ML+lab models screening out diabetes (59). It is reported that introducing laboratory tests, such as urine glucose, LDL-c, and triglyceride increased the predictive accuracy of diabetes predictive models by LR (45, 60), which seemed to be consistent with our findings in this study. Notably, the detection costs were decreased in the ML+FPG model and increased greatly in the ML+2hPG and ML+HbA1c models compared with the ML model, due to the testing of FPG costing much less than the testing of 2hPG and HbA1c (61). Moreover, our results indicated that the ML+FPG model had lower average detection costs compared with the NCDRS+FPG model. These results implied that the ML+FPG model had appreciable advantages in predictive accuracy and the lowest costs among the ML+lab models, the NCDRS+FPG model, and the ML model. The FPG test was most often used by health care professionals in clinical practice for it is convenient and relatively inexpensive. Therefore, the ML+FPG model was the most suitable for primary care among the ML+lab models, for its considerable predictive accuracy, low costs, and easily-accessibility, and was potentially to become a new screening strategy for diabetes in primary care with notable advantages in efficiency, economy, and convenience.

Additionally, SHAP analysis was used to perform a *post hoc* analysis of the model with all available features in the present study. Our data indicated that some predictors, including RPR, Age, SBP, WC, WHR, and BMI, were of significant value in the ML model, which is consistent with previous studies that these are important predictive factors in diabetes predictive models developed by LR and gradient boosting. Typically, the resting pulse rate is often used as an alternative to resting heart rate measurements. Increasing evidence suggested that resting heart rate was associated with type 2 diabetes (62, 63), and recent studies revealed close relevance of resting heart rate and diabetes (64, 65). Detailedly, resting heart rate is an indicator of sympathetic activation, which inhibits the insulin secretion from the pancreas and sympathetic overactivity can impair glucose uptake in skeletal muscle by inducing vasoconstriction and reducing skeletal muscle blood flow (66). These mechanisms might explain the association between RPR and diabetes. The years of cell phone use seemed relevant to age in our present study and are reported to be closely related with socioeconomic status, which might be an important explanations for the close relevance between the years of cell phone use and diabetes (67). Additionally, we identified several predictors, such as sleep duration and wake time, which are reported to be closely associated with FPG and/or HbA1c by multiple linear regression analysis, implying that more attention should be paid to these predictors in the prevention and management of diabetes (68, 69). Furthermore, our results indicated that WC, a useful measure of central obesity, was more important in predicting diabetes compared with BMI in our ML model, which seems consistent with previous studies that WC is considered to be a more reliable measure of fat distribution and closely related with diabetes (70). It is reported that Asians including the Chinese population have more central obesity but less generalized obesity defined by high BMI. These results suggest that we should pay more attention to central obesity in the prevention, screening, and management of diabetes in the Chinese population.

Moreover, our SHAP analysis by taking two cases randomly for example identified different individualized predictors for the two selected cases, suggesting that the cutting-edge SHAP analysis in our ML model was able to screen out the crucial predictors individually for the subjects. The personalized predictors screened out would be helpful for the subject tested to get advice and take measures more accurately and precisely to prevent or treat diabetes (71).

It should be noted that we were unable to determine the detection costs and potential complication costs in the real world in the present study, although we attempted to estimate the costs using rewarding methods reported previously and adjust the costs based on economic and medical conditions in China. These findings require verification by further studies and it should be interpreted cautiously. Additionally, our data set was obtained from Han Chinese population in Hubei Province, which is located in central China, and the generalizability of our models and the findings need further testing with data from more regions and ethnic groups. Noteworthy, we had aimed to preclude the effects of medications as much as possible originally and we made great efforts to exclude the effects of antidiabetic agents on diabetes determination. Regretfully, we were not able to exclude the effects of other medications including those may affect RPR due to the missing data. In addition, the features employed in the models (e.g., RPR) were closely related with diabetes but not always play causal roles in the development of diabetes. Thus, it should be noted cautiously while interpreting the models, the importance of features, and the relationship between/among them, and further research is necessary to confirm the prevention and intervention strategies to take accordingly. Moreover, it would be of great help for us to further iterate models with new algorithms based on the newly inputted data to improve the predictive performance and generalizability of the models in the future.

Notwithstanding these limitations, the ML model developed for diabetes screening in community care had good predictive accuracy and less average detection costs compared with the NCDRS. The ML+FPG model created for diabetes screening in primary care achieved higher predictive accuracy and lower detection costs than the ML model and NCDRS+FPG model. Thus, we tentatively put forward that the ML-augmented algorithm might have the potential to become an efficient and practical tool for diabetes screening in community and primary care settings.

## Data availability statement

The raw data supporting the conclusions of this article will be made available by the authors, without undue reservation.

## Ethics statement

Written informed consent was obtained from the individual(s) for the publication of any potentially identifiable images or data included in this article.

## Author contributions

LLC, XH, and XL contributed to the study design. LLC, XH, TZ, JZ, JM, ST, HZ, HH, and WZ contributed to the data acquisition and data collection. LLC, XL, XH, WZ, QZ, and PW contributed to the data analysis and the preparation of the manuscript. XL, LC, and WZ contributed to checking and modifying code related to ML algorithms. LLC and XH were responsible for the data interpretation and modifying the manuscript. All the authors have critically read the manuscript and approved the submitted version.

## Funding

This research was supported by grants from the National Natural Science Foundation of China (81800762, 82173517, 81770843, 82170822, and 82000366) and the Ministry of Science and Technology of the People’s Republic of China (2016YFC0901200 and 2016YFC0901203).

## Acknowledgments

We thank all participants, partner hospitals, and all staff for their contributions to this research.

## Conflict of interest

The authors declare that the research was conducted in the absence of any commercial or financial relationships that could be construed as a potential conflict of interest.

## Publisher’s note

All claims expressed in this article are solely those of the authors and do not necessarily represent those of their affiliated organizations, or those of the publisher, the editors and the reviewers. Any product that may be evaluated in this article, or claim that may be made by its manufacturer, is not guaranteed or endorsed by the publisher.
