# Supplementary material for: Development and validation of a machine learning-augmented algorithm for diabetes screening in community and primary care settings: A population-based study
Source: Front Endocrinol (Lausanne). 2022 Nov 28;13:1043919. doi: 10.3389/fendo.2022.1043919 (PMC9742532; doi:10.3389/fendo.2022.1043919)
Supplement: Supplementary file 1 [file DataSheet_1.docx]

***Supplementary materials***

**
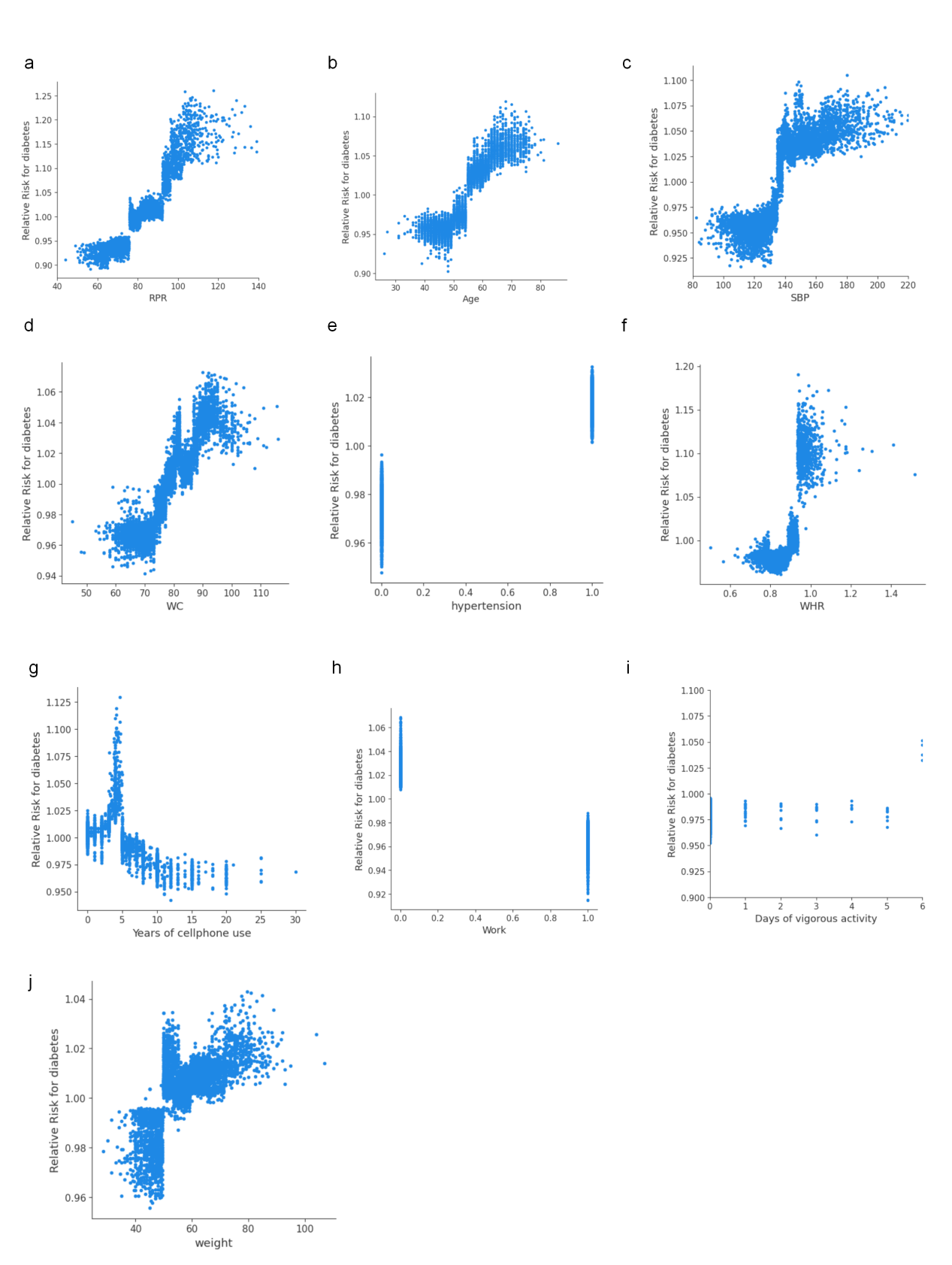
**

**Supplementary Figure 1. The relative risk of diabetes of top-10 important features.** Abbreviations: RPR, resting pulse rate; WHR, waist-to-hip ratio; SBP, systolic blood pressure; WC, waist circumference; WHtR, waist-to-height ratio.


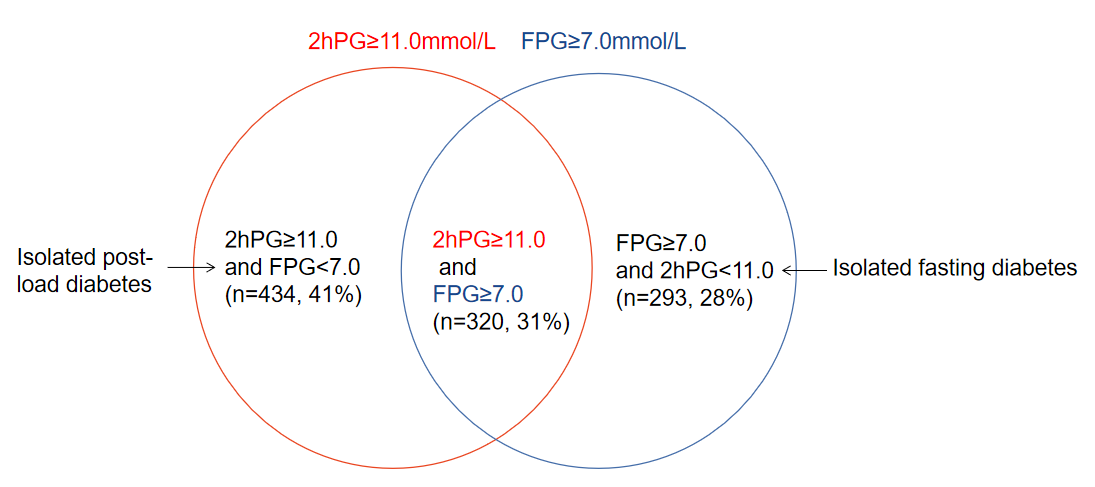


**Supplementary Figure 2. FPG and 2hPG levels in individuals with diabetes.** Abbreviations: FPG, fasting plasma glucose; 2hPG, OGTT-2h post-load plasma glucose.


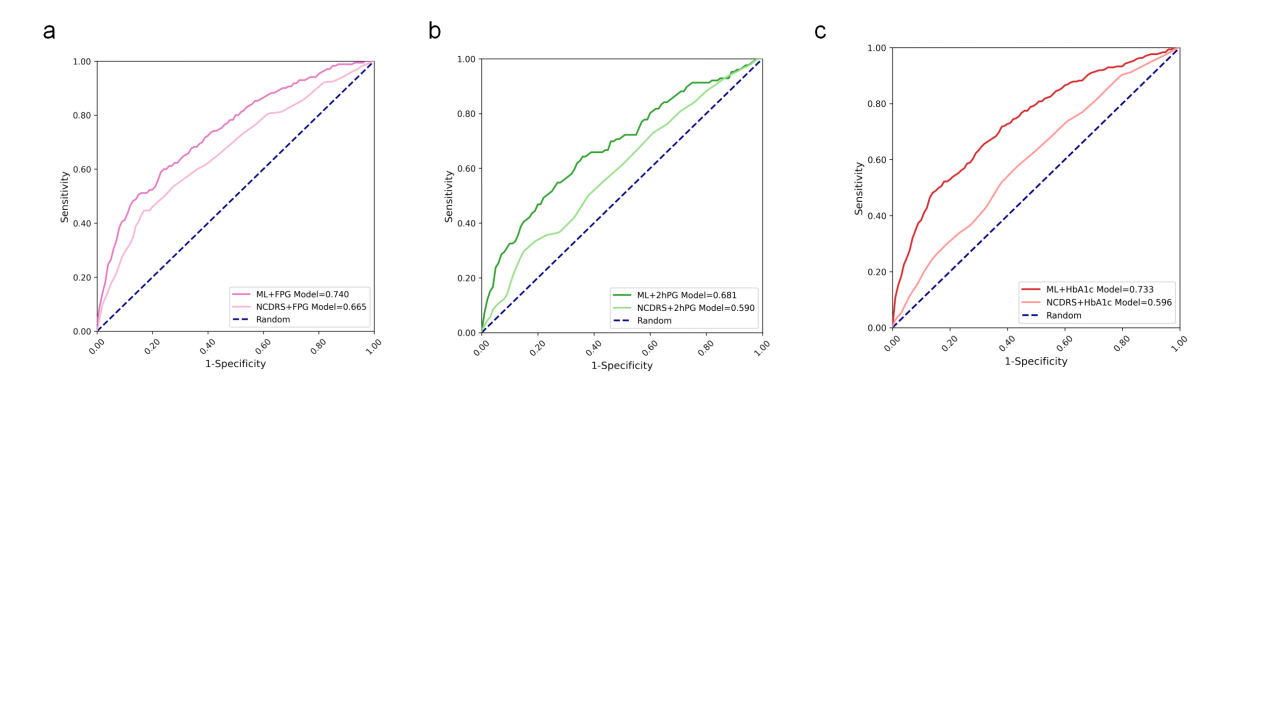


**Supplementary Figure 3. Comparisons of model performance between ML+lab and NCDRS+lab models.** a, Compared the ROC curves between the ML+FPG model and NCDRS+FPG model in the individuals with seemingly normal FPG levels, P< 0·05. b, Compared the ROC curves between the ML+2hPG model and NCDRS+2hPG model in the individuals with seemingly normal 2hPG levels, P<0·05. c, Compared the ROC curves between the ML+HbA1c model and NCDRS+HbA1c model in the individuals with seemingly normal HbA1c levels, P< 0·05.

**
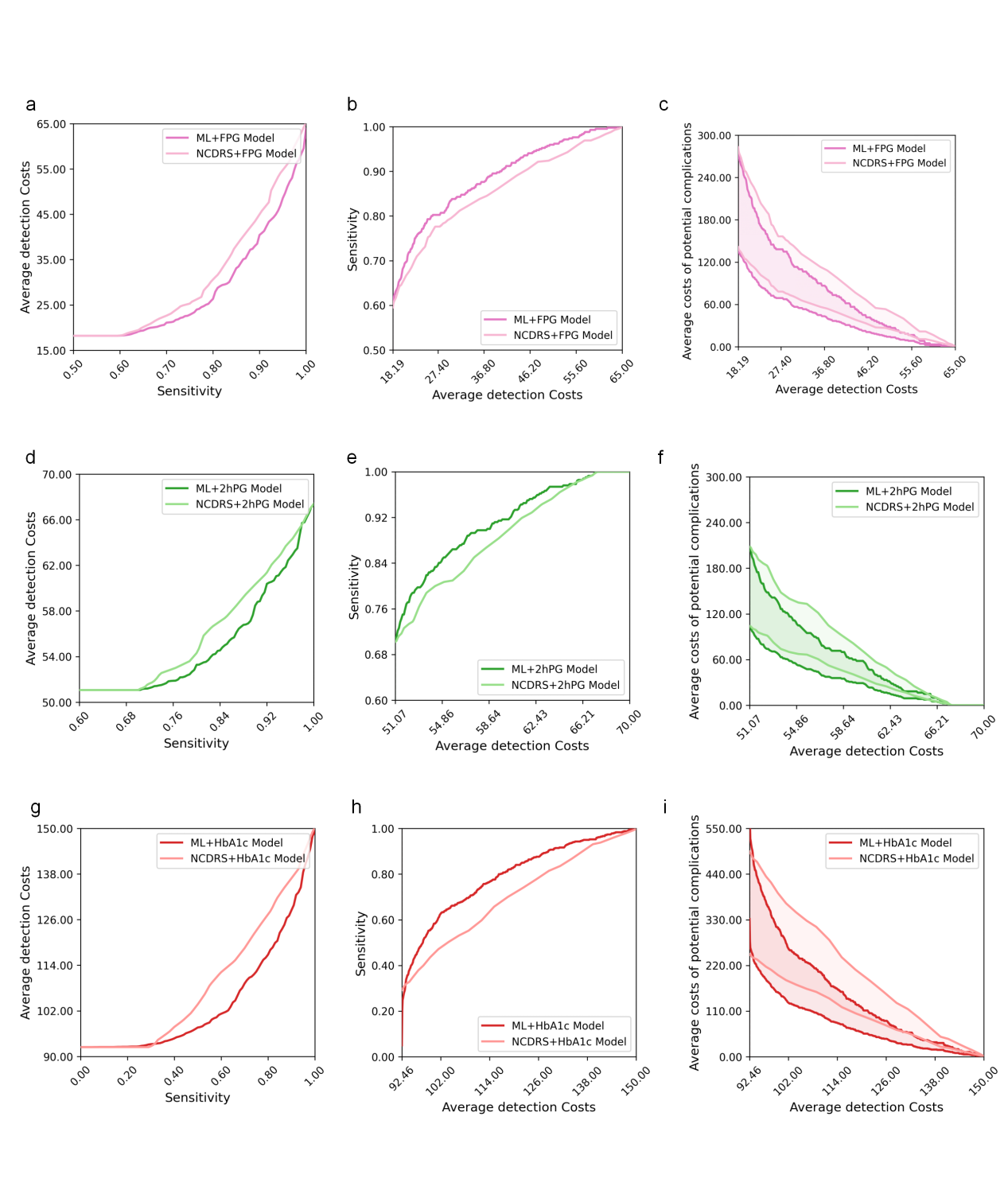
**

**Supplementary Figure 4. Comparisons of health economic costs between ML+lab and NCDRS+lab models.** a, Compared the average detection costs between the ML+FPG model and NCDRS+FPG model at a different level of sensitivity. b-c, Compared the sensitivity (b) and average costs of potential complications for 15 years (c) between the ML+FPG model and NCDRS+FPG model at a different level of average detection costs. d, Compared the average detection costs between the ML+2hPG model and NCDRS+2hPG model at a different level of sensitivity. e-f, Compared the sensitivity (e) and average costs of potential complications for 15 years (f) between the ML+2hPG model and NCDRS+2hPG model at a different level of average detection costs. g, Compared the average detection costs between the ML+HbA1c model and NCDRS+HbA1c model at a different level of sensitivity. h-i, Compared the sensitivity (h) and average costs of potential complications for 15 years (i) between the ML+HbA1c model and NCDRS+HbA1c model at a different level of average detection costs. Shaded areas represent the range of average costs of potential complications for 15 years.

**
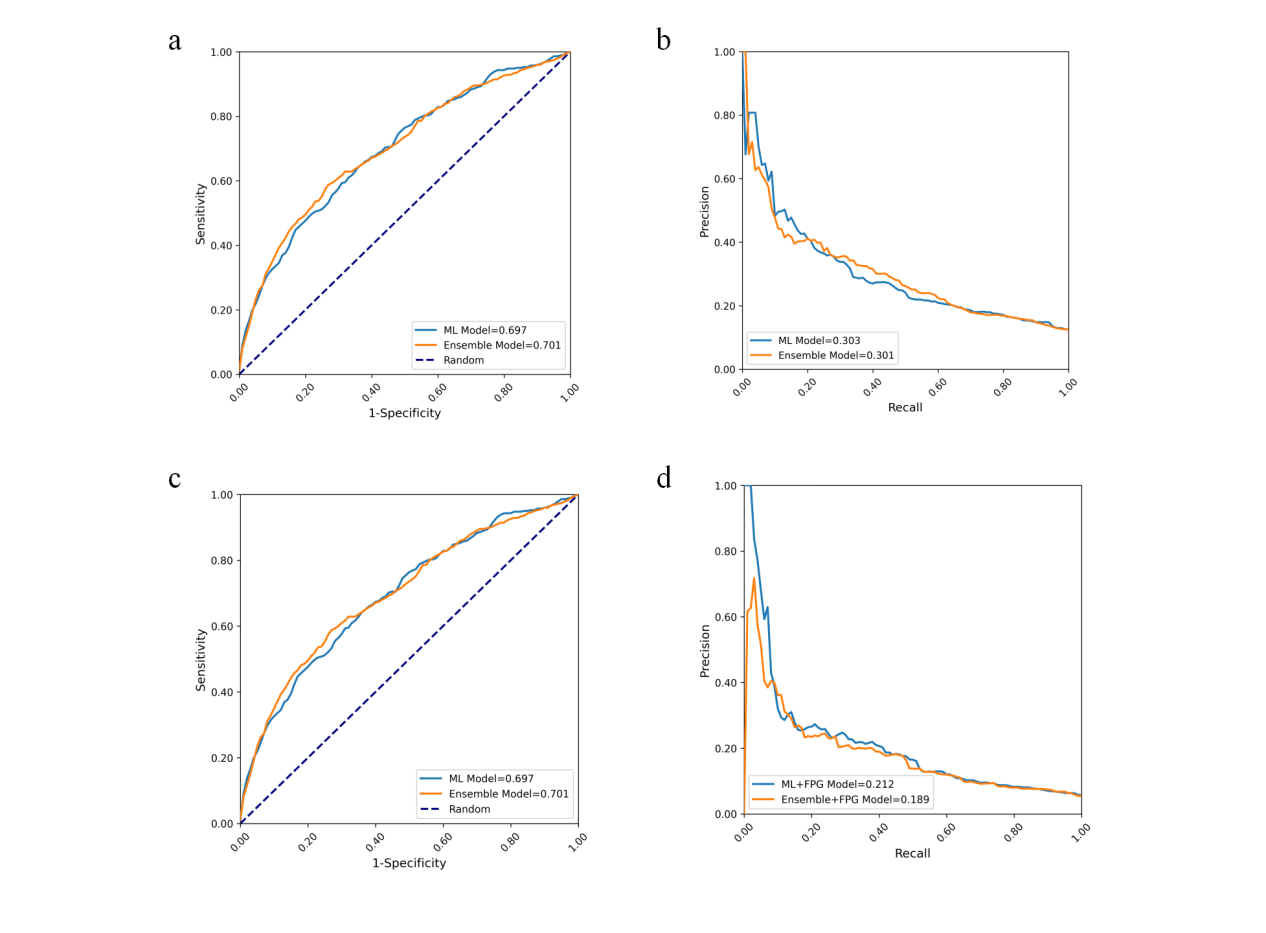
**

**Supplementary Figure 5. Performance comparison of ML model and ML+FPG model on using LGBM algorithm and ensemble algorithm.** a-b, Compared the ROC (a) and PR (b) curves between the ML model and Ensemble model, AUC, P>0·05. c-d, Compared the ROC (c) and PR (d) curves between the ML+FPG model and Ensemble+FPG model, AUC, P>0·05.

|  | Numbers (%) of missing data | Complete cases | | | Imputation data | | |
| --- | --- | --- | --- | --- | --- | --- | --- |
|  |  | Distribution | OR (95% CI) | P | Distribution | OR (95% CI) | P |
| Education^a^ | 68 (1·3) |  |  |  |  |  |  |
| Illiteracy |  | 9·6% | reference |  | 9·5% | reference |  |
| Primary |  | 38·7% | 1.31 (0.97-1.80) | 0.090 | 39·0% | 1.41 (1.05-1.92) | 0.028 |
| Junior |  | 38·9% | 0.89 (0.65-1.24) | 0.487 | 38·9% | 0.93 (0.68-1.28) | 0.629 |
| Senior |  | 11·3% | 0.60 (0.39-0.92) | 0.020 | 11·1% | 0.58 (0.38-0.89) | 0.012 |
| Junior college |  | 1·5% | 0.92 (0.39-1.93) | 0.843 | 1·5% | 0.89 (0.38-1.85) | 0.772 |
| Diabetes family history^a^ | 630 (12·5) |  |  |  |  |  |  |
| No |  | 98·7% | reference |  | 98·9% | reference |  |
| Yes |  | 1·3% | 0.90 (0.35-1.96) | 0.818 | 1·1% | 0.86 (0.33-1.87) | 0.736 |
| Work^a^ | 302 (6·0) |  |  |  |  |  |  |
| No |  | 62·1% | reference |  | 60·5% | reference |  |
| Yes |  | 37·9% | 0.64 (0.53-0.77) | <0·001 | 39·5% | 0.63 (0.53-0.75) | <0.001 |
| Years of cellphone use^b^ | 258 (5·1) | 4·4 | 0.96 (0.94-0.98) | <0·001 | 4·4 | 0.96 (0.94-0.98) | 0.001 |
| Wake time^b^ | 414 (8·2) | 6·5 | 0.96 (0.87-1.06) | 0.445 | 6·5 | 0.97 (0.88-1.07) | 0.519 |
| Daytime sleep duration, h^b^ | 402(8·0) | 0·2 | 1.02 (0.94-1.09) | 0.651 | 0·2 | 1.00 (0.93-1.07) | 0.939 |
| RPR, bpm^b^ | 69 (1·4) | 81·2 | 1.04 (1.03-1.04) | <0·001 | 81·2 | 1.04 (1.03-1.04) | <0·001 |
| SBP, mmHg^b^ | 11 (0·2) | 138·0 | 1.02 (1.01-1.02) | <0·001 | 138·0 | 1.02 (1.01-1.02) | <0·001 |
| DBP, mmHg^b^ | 12 (0·2) | 79·7 | 1.02 (1.01-1.03) | <0·001 | 79·7 | 1.02 (1.01-1.03) | <0·001 |
| Weight, kg^b^ | 36 (0·7) | 57·6 | 1.02 (1.01-1.03) | <0·001 | 57·6 | 1.02 (1.01-1.03) | <0·001 |
| Height, m^b^ | 33 (0·7) | 156·8 | 1.00 (0.98-1.01) | 0.477 | 156·8 | 1.00 (0.98-1.01) | 0.415 |
| WC, cm^b^ | 35 (0·7) | 78·3 | 1.03 (1.02-1.04) | <0·001 | 78·3 | 1.03 (1.02-1.04) | <0·001 |
| HC, cm^b^ | 38 (0·8) | 90·9 | 1.01 (1.00-1.03) | 0.032 | 90·9 | 1.01 (1.00-1.03) | 0.040 |

**Supplementary Table 1. Comparison of distributions of variables, and odds ratio of variables for diabetes between complete cases and multiple imputation data in the training set.** Notes: a, Data (continuous variables) of distribution are expressed as mean; b, Data (categorical variables) of distribution are expressed as percentage (%); ORs for diabetes were calculated by univariable regression analyses.

Abbreviations: OR, odds ratio; CI, confidence interval; RPR, resting pulse rate; SBP, systolic blood pressure; DBP, diastolic blood pressure; WC, waist circumference; HC, hip circumference.

|  | Numbers (%) of missing data | Complete cases | | | Imputation data | | |
| --- | --- | --- | --- | --- | --- | --- | --- |
|  |  | Distribution | OR (95% CI) | P | Distribution | OR (95% CI) | P |
| Education^a^ | 51 (1·5) |  |  |  |  |  |  |
| Illiteracy |  | 9·7% | reference |  | 9·6% | reference |  |
| Primary |  | 37·4% | 0.92 (0.65-1.33) | 0.660 | 37·8% | 1.06 (0.75-1.51) | 0.750 |
| Junior |  | 38·4% | 0.65 (0.45-0.94) | 0.019 | 38·3% | 0.68 (0.48-0.99) | 0.038 |
| Senior |  | 13·2% | 0.72 (0.47-1.12) | 0.146 | 13·0% | 0.71 (0.46-1.10) | 0.123 |
| Junior college |  | 1·3% | 1.23 (0.48-2.79) | 0.640 | 1·2% | 1.21 (0.47-2.74) | 0.669 |
| Diabetes family history^a^ | 450(13·4) |  |  |  |  |  |  |
| No |  | 98·7% | reference |  | 98·8% | reference |  |
| Yes |  | 1·3% | 0.79 (0.24-1.99) | 0.657 | 1·2% | 0.80 (0.24-2.02) | 0.675 |
| Work^a^ | 171 (5·1) |  |  |  |  |  |  |
| No |  | 62·9% | reference |  | 61·3% | reference |  |
| Yes |  | 37·1% | 0.68 (0.54-0.85) | 0·001 | 38·7% | 0.71 (0.57-0.88) | 0.002 |
| Years of cellphone use^b^ | 192 (5·7) | 4·3 | 0.96 (0.94-0.99) | 0.010 | 4·3 | 0.97 (0.94-0.99) | 0.019 |
| Wake time^b^ | 287 (8·5) | 6·5 | 0.91 (0.80-1.03) | 0.135 | 6·5 | 0.92 (0.81-1.04) | 0.171 |
| Daytime sleep duration, h^b^ | 286 (8·5) | 0·3 | 1.10 (1.03-1.17) | 0.003 | 0·3 | 1.09 (1.02-1.15) | 0.011 |
| RPR, bpm^b^ | 45(1·3) | 81·1 | 1.04 (1.03-1.05) | <0·001 | 80·8 | 1.04 (1.03-1.05) | <0·001 |
| SBP, mmHg^b^ | 1 (0) | 137·7 | 1.01 (1.01-1.02) | <0·001 | 137·3 | 1.01 (1.01-1.02) | <0·001 |
| DBP, mmHg^b^ | 1(0) | 79·6 | 1.02 (1.01-1.03) | <0·001 | 79·5 | 1.02 (1.01-1.03) | <0·001 |
| Weight, kg^b^ | 23 (0·7) | 57·5 | 1.02 (1.01-1.03) | <0·001 | 57·3 | 1.02 (1.01-1.03) | <0·001 |
| Height, m^b^ | 25 (0·7) | 156·8 | 0.99 (0.98-1.01) | 0.335 | 156·7 | 0.99 (0.98-1.01) | 0.406 |
| WC, cm^b^ | 27 (0·8) | 78·2 | 1.03 (1.02-1.04) | <0·001 | 78·0 | 1.03 (1.02-1.04) | <0·001 |
| HC, cm^b^ | 26 (0·8) | 90·8 | 1.02 (1.00-1.03) | 0.048 | 90·6 | 1.02 (1.00-1.03) | 0.052 |

**Supplementary Table 2.** **Comparison of distributions of variables, and odds ratio of variables for diabetes between complete cases and multiple imputation data in the testing set.** Notes: a, Data (continuous variables) of distribution are expressed as mean; b, Data (categorical variables) of distribution are expressed as percentage (%); ORs for diabetes were calculated by univariable regression analyses.

Abbreviations: OR, odds ratio; CI, confidence interval; RPR, resting pulse rate; SBP, systolic blood pressure; DBP, diastolic blood pressure; WC, waist circumference; HC, hip circumference.

|  | Library | Hyperparameter |
| --- | --- | --- |
| LGBM | LightGBM (https://github.com/microsoft/LightGBM) | "num_leaves": 21,  "max_bin": 16,  "max_depth": 196,  "learning_rate": 0.01967691580047528,  "lambda_l1": 0.0005,  "lambda_l2": 0,  "feature_fraction": 0.2421253618009045,  "min_data_in_bin": 4,  "bagging_fraction": 0.37134465281597856,  "bagging_freq": 0,  "path_smooth": 0 |
| ANN | Pytorch (https://pytorch.org/) | "lr": 0.0007030852815858443,  "weight_decay": 0.01,  "batch_size": 55,  "enable_lr_scheduler": 1,  "opt": "RMSprop",  "n_channels": 221,  "n_layers": 5,  "dropout": 1,  "bn": 0,  "activate": "Tanh", |
| KNN | sklearn.neighbors.KNeighborsClassifier (https://scikit-learn.org/stable/modules/generated/sklearn.neighbors.KNeighborsClassifier.html) | "n_neighbors": 28,  "leaf_size": 13, "weights": "distance", "p": 1 |
| CDKNN | (https://github.com/ngphubinh/CDNN) | n_neighbors=20, distance_metric="euclidean" |
| RF | sklearn.ensemble.RandomForestClassifier (https://scikit-learn.org/stable/modules/generated/sklearn.ensemble.RandomForestClassifier.html) | "n_estimators": 159,  "max_depth": 4,  "min_samples_split": 4,  "min_samples_leaf": 5,  "criterion": "entropy", "min_weight_fraction_leaf": 0.1, |
| SVM | sklearn.svm.SVC (https://scikit-learn.org/stable/modules/generated/sklearn.svm.SVC.html) | "C": 3.100100315037076,  "kernel": "rbf", "degree": 3, "gamma": "auto" |
| LR | sklearn.linear_model.LogisticRegression (https://scikit-learn.org/stable/modules/generated/sklearn.linear_model.LogisticRegression.html) | "C": 1.0019981646171054,  "penalty": "l2",  "dual": True,  "solver": "liblinear" |

**Supplementary Table 3. The hyperparameter in the models with seven machine learning algorithms.**

Abbreviation: LGBM, light gradient boosting machine; ANN, Artificial Neuron Network; KNN, K-nearest neighbors algorithm; CDKNN, Centroid-Displacement-based-k-NN; RF, Random Forest; SVM, Support Vector Machine; LR, Logistic Regression.

|  | Proportion requiring confirmatory test | Costs of screening tests (CNY) | Costs of Confirmatory tests (CNY) | Average detection costs per participant (CNY) |
| --- | --- | --- | --- | --- |
| ML model | TP+FP/N | - | 60·95 (OGTT) | 60·95*(TP+FP)/N |
| NCDRS |  |  |  |  |
| ML+FPG model | (TP+FP-n1)/N | 18·19 (FPG) | 51·06 (2hPG) | [18·19*(N) + 51·06*(TP+FP-n1)]/N |
| NCDRS+FPG model |  |  |  |  |
| ML+2hPG model | (TP+FP-n2)/N | 51·06 (2hPG) | 18·19 (FPG) | [51·06*(N) + 18·19*(TP+FP-n2)]/N |
| NCDRS+2hPG model |  |  |  |  |
| ML+HbA1c model | (TP+FP)/N | 92·46 (HbA1c) | 60·95 (OGTT) | [92·46*N+60·95*(TP+FP)]/N |
| NCDRS+HbA1c model |  |  |  |  |

**Supplementary Table 4. The proportion requiring confirmatory test and average detection costs estimation.** Abbreviations: TP, True positive cases; FP, false positive cases; N, the number of participants in the testing set; n1, the number of individuals with FPG ≥ 7mmol/l in the testing set; n2, the number of individuals with 2hPG ≥ 11·1mmol/l in the testing set.

|  | Average costs of potential complications per participant per year (CNY) | |
| --- | --- | --- |
| 5 years | (341 to 567) | * (FN/N) |
| 10 years | (1302 to 2555) |  |
| 15 years | (2802 to 5611) |  |
| 20 years | (4428 to 8212) |  |
| 25 years | (5258 to 9132) |  |

**Supplementary Table 5. The average costs of potential complications estimation.** Abbreviations: FN, false negative cases in the testing set; N, the number of participants involved in the testing set.

|  | LGBM | ANN | RF | SVM | LR | KNN |
| --- | --- | --- | --- | --- | --- | --- |
| LGBM | - | - | - | - | - | - |
| ANN | 0·67 | - | - | - | - | - |
| RF | 0.073 | 0.46 | - | - | - | - |
| SVM | 0.00044 | 0.00034 | 0.013 | - | - | - |
| LR | 0.017 | 0.014 | 0.19 | 0.086 | - | - |
| KNN | 1.2e-09 | 1.9e-08 | 3.3e-07 | 0.0058 | 0.0002 | - |
| CDKNN | 7.6e-12 | 7.6e-12 | 3.8e-09 | 3.4e-06 | 5.6e-08 | 0.049 |

**Supplementary Table 6. Comparison of statistical differences in AUC (P values) of five algorithms.** The P values for AUCs comparison were calculated using Delong tests. Abbreviations: LGBM, light gradient boosting machine; ANN, Artificial Neuron Network; RF, Random Forest; SVM, Support Vector Machine; LR, Logistic Regression; KNN, K-nearest neighbors algorithm ; CDKNN, Centroid-Displacement-based-k-NN; AUC, area under the receiver operating characteristic curve (ROC).

|  | ML model | NCDRS |
| --- | --- | --- |
| ML model | - | - |
| NCDRS | 2.6e-09 | - |
| ADART | 4.2e-09 | 0.098 |

**Supplementary Table 7. Comparison of statistical differences in AUC (P values) of ML model, NCDRS, ADART.** The P values for AUCs comparison were calculated using Delong tests. Abbreviations: NCDRS, New Chinese Diabetes Risk Score; ADART, ADA (America Diabetes Association) risk test; AUC, area under the receiver operating characteristic curve.
